# A phantom based evaluation of the dose prediction and effects in treatment plans, when calculating on a direct density CT reconstruction

**DOI:** 10.1002/acm2.12824

**Published:** 2020-03-16

**Authors:** Veronika Flatten, Alexandra Friedrich, Rita Engenhart‐Cabillic, Klemens Zink

**Affiliations:** ^1^ Department of Radiotherapy and Radiooncology University Medical Center Giessen‐Marburg Marburg Germany; ^2^ Institute of Medical Physics and Radiation Protection University of Applied Sciences Giessen Germany; ^3^ Department of Radiotherapy RNS Gemeinschaftspraxis Wiesbaden Germany; ^4^ MIT Marburg Ion Beam Therapy Center Marburg Germany

**Keywords:** CT reconstruction algorithm, DirectDensity, electron density reconstruction, image reconstruction

## Abstract

In radiation therapy, a Computed Tomography (CT) image is needed for an accurate dose calculation. To allow such a calculation, the CT image values have to be converted into relative electron densities. Thus, standard procedure is to calibrate the CT numbers to relative electron density (RED) by using a phantom with known composition inserts. This calibration curve is energy and CT dependent, therefore most radiotherapy CT acquisitions are obtained with 120 kVp, as each tube voltage needs an additional calibration curve. The commercially available DirectDensity^TM^ (DD) reconstruction algorithm presents a reconstruction implementation without any dependence on the tube voltage. In comparison, it allows a calibration curve that is directly proportional to the RED, reducing the need of more than one calibration curve. This could potentially optimize CT acquisitions and reducing the dose given to the patient. Three different phantoms were used to evaluate the DirectDensity^TM^ algorithm in simple and anthropomorphic geometries, as well as setups with metal implants. Scans with the DD algorithm were performed for 80, 100, 120, and 140 kVp. As reference a scan with the standard 120 kVp scan was used. Radiotherapy photon plans were optimized and calculated on the reference image and then transferred to the DD images, where they were recalculated. The dose distributions obtained this way were compared to the reference dose. Differences were found mainly in pure air and high density materials such as bones. The difference of the mean dose was below 0.7%, in most cases below 0.4%. No indication was found that the algorithm is corrupted by metal inserts, enabling the application for all clinical cases. This algorithm offers more variability in CT parameters for radiation therapy and thus a more personalized image acquisition with a high image quality and a lower dose exposure at a robust clinical workflow.

## 1 INTRODUCTION

In radiation therapy, the Computed Tomography (CT) images provide the essential patient geometry and electron density information needed not only to delineate the tumor and organs at risk but also to calculate dose. Beforehand, a relation between CT numbers and relative electron density (RED) or mass density (MD) has to be established in the form of a calibration curve in the treatment planning system (TPS) as described by e.g., Schneider et al.^1^. In the common case, the CT pixel values are given in Hounsfield units (HU) and depend on the tube voltage. Thus, a calibration curve for each tube voltage is needed.^1^, ^2^ Some clinics feature more than the standard 120 kVp calibration curve e.g., an additional 80 kVp curve for pediatric patients. This potentially reduces the CT dose for the patient or offers a higher contrast‐to‐noise ratio but increases the amount of work while the workflow robustness is decreased by the additional selection of the calibration curves. Other clinics only implement the standard 120 kVp curve for all scans ignoring the potential benefit of a higher signal‐to‐noise ratio or a decreased dose exposure that could be achieved with an optimized tube voltage.^3^, ^4^, ^5^

The commercially available reconstruction algorithm ‘DirectDensity’ (DD) (Siemens Healthcare, Erlangen, Germany) promises a feasible workflow as it constructs the RED information directly from the raw CT data.^6^ DD‐CT images obtained this way are independent of the tube voltage once the calibration curve is implemented in the TPS.

Van der Heyden showed in a patient study for 33 patients that the mean dose difference between HU based dose calculated plans and those calculated on the DD image sets were found to be smaller than 1%.^7^ In this study, the relative mean dose difference was evaluated for the planning target volume (PTV) and the organs at risk (OAR), as well as the relative difference in the volume receiving 95% of the prescribed dose were evaluated. These differences were also found to be less than 1%. These results are in agreement with the evaluation presented by Ritter.^6^ Nevertheless, both works do not differentiate for cases that provide high uncertainties in imaging and dose calculation, such as air or metal cavities.

The present study analyzes the effect of the DD images on the dose distributions for a phantom with materials with high, medium and low density, exemplary for the density range present in the human body. As analyzing tools, a simple dose difference is accompanied by dose profiles, allowing a distinct analysis where the differences occur.

Additionally, CT scans with metal implants are compared as well as the combination of the DD algorithm with a metal reduction algorithm,^8^ to evaluate if DD can be used in all clinical cases. Otherwise, for metal implants a standard conversion curve would be needed, revoking the easy setup and robust usability when implementing the DD algorithm.

Finally, the potential CT dose reduction benefits that could be utilized when dose acquisition parameters (e.g., the tube voltage) are optimized for image quality, are presented.

## 2 MATERIALS AND METHODS

### 2.A DirectDensity reconstruction

To understand the procedure behind the DirectDensity images and understand problematic setups and cases, a brief introduction of the algorithm is given. A more detailed description is given by Ritter.^6^

**Figure 1:**
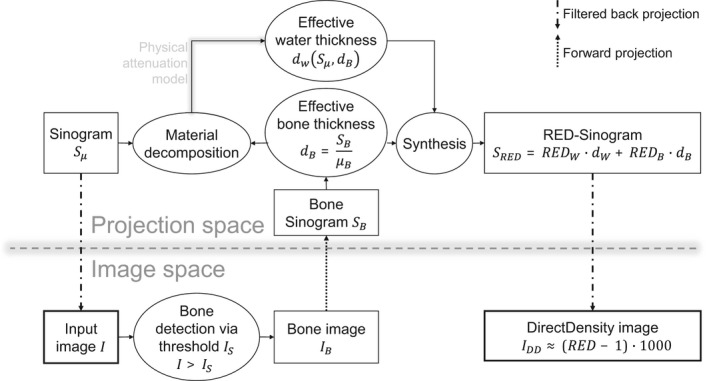
A flowchart Ritter^6^ that displays the main steps of the DirectDensity algorithm. The steps can be divided in the ones taking place in the image space and the ones performed in the projection space.

Figure 1 describes the main steps in the algorithm. First, a single energy CT image (I) is needed for the DirectDensity reconstruction. By applying an attenuation threshold (I_S_), a bone image (I_B_) is generated. In the projection plane, a model based material decomposition is applied to the original sinogram (S_µ_), separating all materials present in the human body into either water or bone. With an underlying physical attenuation model, the effective water thickness (d_W_) for all materials can be obtained. The combination of the effective bone thickness (d_B_) gained from the information of the bone sinogram (S_B_) and the effective water thickness (d_W_) together with the RED of water and bone gives an electron density line integral (S_RED_). Thus, in a final step a RED proportional image (I_DD_) via filtered back projection of the RED sinogram is gained.

The connection between the image values of the final DD image and the RED is given in eq. (1).^6^

(1) $$I_{\mathrm{DD}}\;\approx \;\left(RED-1\right)*1000\Rightarrow \underset{Implemented\;calibration\;inthis\;work}{\underbrace{I_{\mathrm{DD}}=\left(RED-0.994\right)*1000}}$$

### 2.B Density calibration

**Figure 2:**
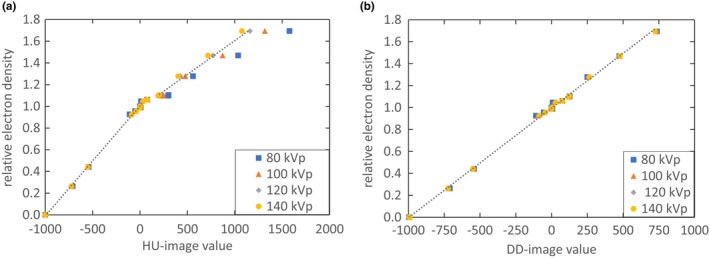
The calibration curves to convert the CT image values into RED for the HU reconstruction (left) and the DD reconstruction (right) for all four tube voltages. The values were obtained with inserts of the Gammex 467 Tissue Characterization Phantom. The dotted linear regression was performed on the 120 kVp data points.

To obtain the RED information from the CT scan, calibration curves for 80, 100, 120, and 140 kVp tube voltage were generated with the Gammex 467 Tissue Characterization Phantom (Gammex, Giessen‐Allendorf, Germany) for the HU and the DD reconstruction. All scans were taken with a constant tube current of 250 mAs on the SOMATOM Confidence (Siemens Healthcare, Erlangen, Germany). The reconstruction with 3 mm slice thickness was executed with the B40s and E30s convolution filters. For the calibration, the mean CT value of each tissue mimicking insert was plotted against the RED of the material. The mean CT value for each insert was obtained in ImageJ (v1.50i, National Institutes of Health, Bethesda, USA). The CT values were averaged over a sphere of 2 cm diameter, centered in the middle of the insert. As the calibration curves in Fig. 2 show, the energy dependence vanishes when the DD reconstruction is applied. The right panel displays this implemented calibration given in eq. (1).

### 2.C Study setup

**Figure 3:**
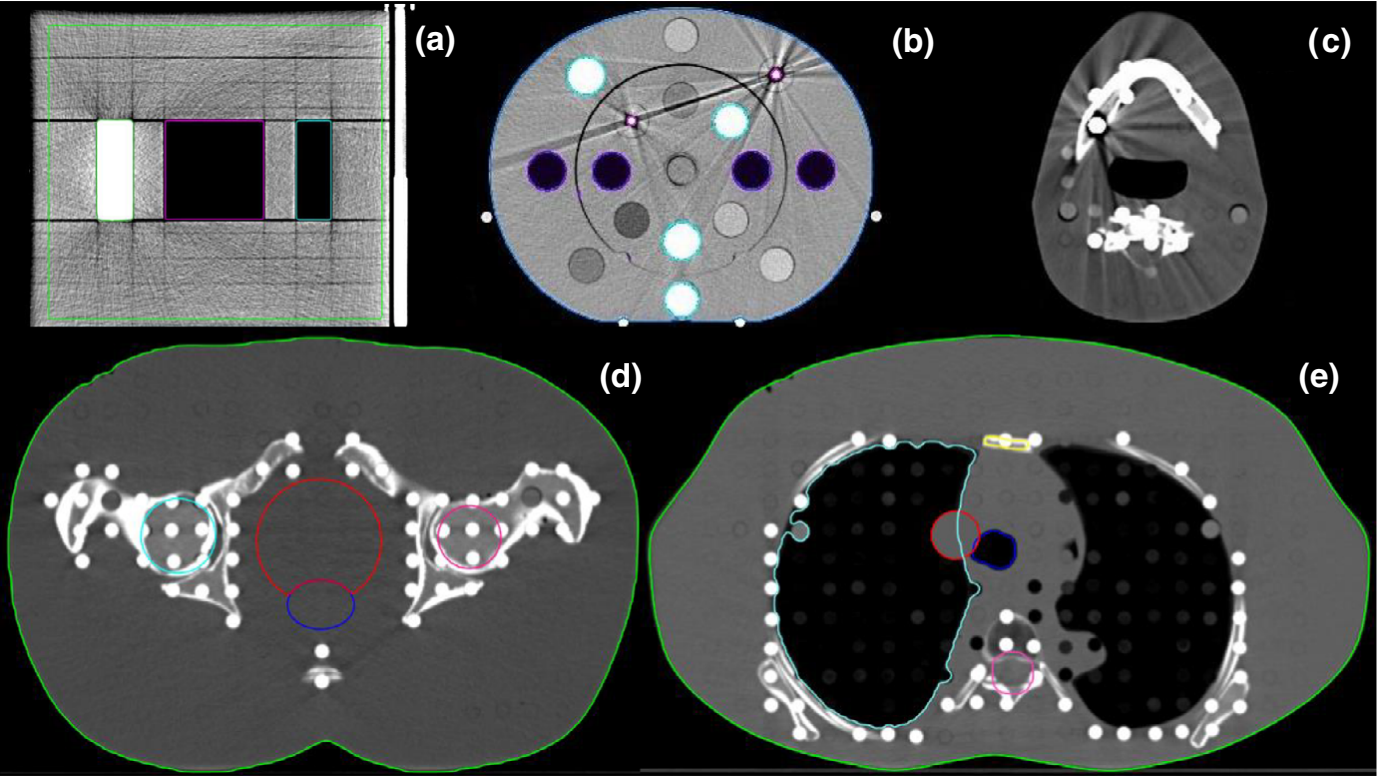
Phantom overview: Panel (a) shows the IMRT phantom in a transverse view. Panel (b) displays the CBCT phantom with its inserts. In panel (c) the metal insert is included in the ART phantom head. Panels (d) and (e) show the ART phantom in a transverse slice in the abdominal and thoracic region.

All phantoms displayed in Fig. 3 were scanned with the SOMATOM Confidence CT. In general, images for 80, 100, 120 kVp, as well as 140 kVp were generated for the E30s (DD) kernel. The reference images (HU) were done with 120 kVp and the B40s kernel, as this represents the standard parameters in the clinical routine. The tube current was set between 140 and 300 mAs, depending on the thickness of the phantom, but held constant for each phantom. Slices of 3 mm thickness were acquired. All scans were imported into Eclipse (version 13.6, Varian Medical Systems, Palo Alto, CA, USA). The contouring was performed on the HU image and then copied to the DD images. The same procedure was followed creating the dose plans: Each plan was optimized and calculated on the 120 kVp HU image and transferred to all DD images. Then, each plan was recalculated holding all beam parameters constant.

The dose was calculated in the TPS Eclipse with the Anisotrop Analytical Algorithm (AAA) (v.13.6.23, Eclipse, Varian Medical Systems, Palo Alto, CA, USA), a 3D pencil beam superposition convolution algorithm. For this algorithm, only RED but not MD needs to be assigned to the CT voxels for a correct dose calculation.^9^, ^10^ Thus, the immediate relation between dose and the DD images which contain RED information is evaluated. The dose calculation grid size was chosen to be 0.125 cm.

To compare the different dose distributions, a dose difference between each plan on the DD reconstruction with the HU dose distribution as reference was created. Because the dose distributions were calculated on identically positioned phantom CT scans, which were performed without moving the phantom, the gamma criteria^11^ which is typically used is not needed. However, passing rates for the dose differences are presented which equal a gamma analysis with 0 mm distance‐to‐agreement and 0.5% dose difference in the region with doses higher than 5% of the maximum dose.

Additionally, dose‐volume histograms (DVH) were used to evaluate dose distributions in specific regions like the PTV and surrounding OARs as contoured in Fig. 3. For the general evaluation, dose profiles were used to further investigate the effect of high and low density materials on the dose distribution.

#### 2.C.1 Setup for the general DD evaluation

To evaluate the dose distribution in simple geometries a slightly modified version of the planar IMRT phantom Gammex 473 (Gammex, Giessen‐Allendorf, Germany), scanned with 250 mAs, was used. The phantom, consisting of solid water slabs, was extended by a cork as well as two plaster inserts as shown in (a) in Fig. 3. These materials were chosen because the RED of plaster is close to typical bone RED, while the cork RED matches lung density. One cavity was left unfilled to also examine effects in air.

To also evaluate a more realistic setup, the anthropomorph male Alderson Radiation Therapy (ART) Phantom (RSD Radiology Support Devices, Long Beach, USA) was additionally investigated. Scans were conducted for the pelvis (scanned with 250 mAs) and thorax region (scanned with 140 mAs) as presented in part (d) and (e) of Fig. 3.

On the IMRT phantom, a single 6 MV open field was planned in Eclipse with a TrueBeam model (Varian Medical Systems, Palo Alto, CA, USA). The plan was computed on the HU image and then copied to all DD images, whereas the assigned monitor units (MU) were held constant. For the Alderson phantom, a 3D conformal and a volumetric modulated arc therapy (VMAT) plan was optimized for a simulated prostate and lung carcinoma treatment.

#### 2.C.2 Setup for metal implants

One of the main advantages of the implementation of the DD algorithm in the clinic is the reduction from many CT calibration curves to only one. Therefore, the effect of metal implants in combination with the DD reconstruction was tested, as the DD reconstruction could define the metal as high density bone due to the applied threshold. If this is the case, an inadequate interpretation of metal could wrongfully influence the reconstruction around these implants. For this study, a third phantom was used. The CBCT Electron Density Phantom (CIRS, Norfolk, USA), as shown in (b) in Fig. 3, which is comparable to the Gammex Tissue Characterization Phantom, was chosen. It has the possibility to insert high density materials like titanium and stainless steel next to bone, adipose and lung tissue equivalents. Using this phantom allows unbiased results because it was not used to produce the calibration curve. The scan was performed with 300 mAs.

For a more anthropomorphic setup, the ART head was scanned with and without a brass insert simulating a dental prosthesis at 200 mAs. As differences due to the artifacts induced by metal ought to appear, an additional reconstruction with a metal reduction algorithm (Siemens Healthcare, Erlangen, Germany) was also evaluated. The iterative Metal Artifact Reduction (iMAR) algorithm reduces the artifacts caused by metal implants via beam hardening correction, sinogram inpainting and frequency split^8^. As most dose calculation algorithms have large uncertainties when calculating in metal, only a RED comparison was performed.

All RED estimations were performed with the presented calibration curves limited to a maximum RED of 1.7. Thus differences for higher RED ought to appear.

#### 2.C.3 CARE dose option

To evaluate the possible dose savings when the DD algorithm is implemented, the tube‐current as well as the voltage were varied. The pelvis and thorax of the ART phantom was scanned again using CARE kV and CARE Dose4D (Siemens Healthcare, Erlangen, Germany). The CARE Dose4D algorithm modulates the tube current on the basis of a topogram^12^ during the scan to reduce dose exposure while maintaining a high image quality without affecting CT numbers. CARE kV on the other hand suggests a probable tube voltage and regulates the tube current to deliver the same Contrast‐to‐Noise‐Ratio. These CARE options changed the CT parameters in such a way that the patient could potentially benefit from a reduced dose exposure without reducing the image quality.

## 3 RESULTS

### 3.A General evaluation of the DirectDensity algorithm

#### 3.A.1 RED comparison

First, the relative electron density of different materials in the IMRT phantom is compared. As the dose computation relies on the right mapping of the RED, differences in the mean RED will most likely result in dose differences. Table 1 gives the mean RED found in the different materials of the IMRT phantom with its standard deviation for all CT reconstructions. It can be observed that the assigned RED in the DD cases is higher the lower the tube voltage. Thus, the tube voltage dependence is not completely vanished.

**Table 1:** Mean RED and its standard deviation for three different materials of the IMRT phantom for all reconstructions.

CT set	$\bar{\text{RED}}$_Plaster_	$\bar{\text{RED}}$_Cork_	$\bar{\text{RED}}$_Air_
HU_120_	1.37 ± 0.01	0.26 ± 0.02	0.04 ± 0.01
DD_80_	1.43 ± 0.06	0.27 ± 0.02	0.03 ± 0.01
DD_100_	1.39 ± 0.02	0.26 ± 0.02	0.03 ± 0.02
DD_120_	1.35 ± 0.03	0.26 ± 0.02	0.02 ± 0.01
DD_140_	1.35 ± 0.01	0.26 ± 0.02	0.02 ± 0.01

#### 3.A.2 Simple geometry

**Figure 4:**
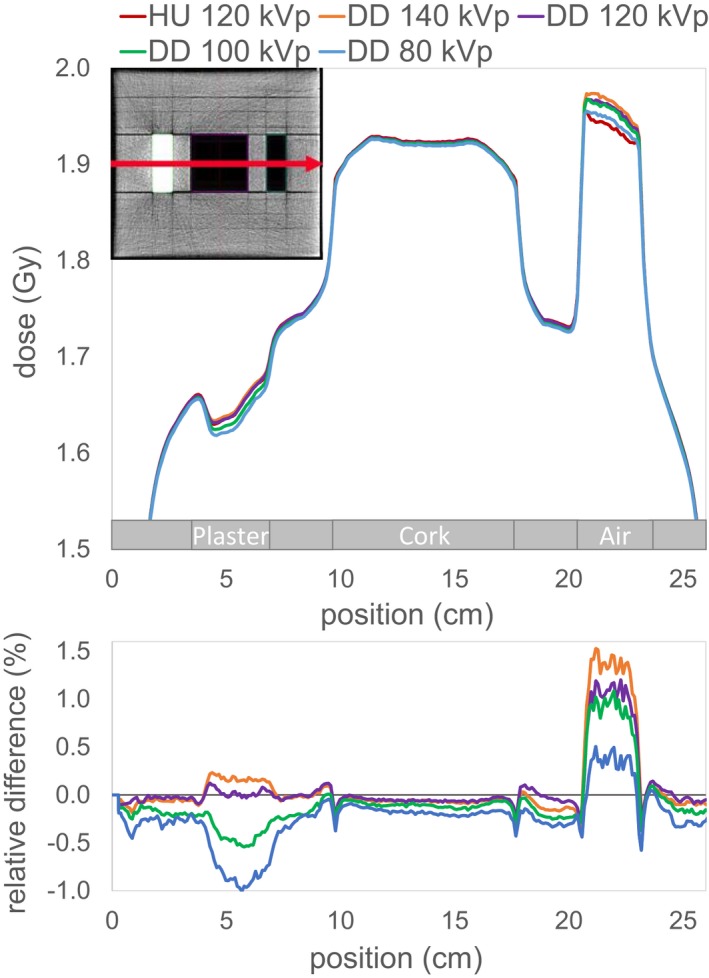
Lateral dose profile along the red line indicated in the upper left corner for the single field irradiated from above for the HU and the four DD reconstructions. The lower plot shows the relative differences of the DD doses compared to the HU dose.

**Figure 5:**
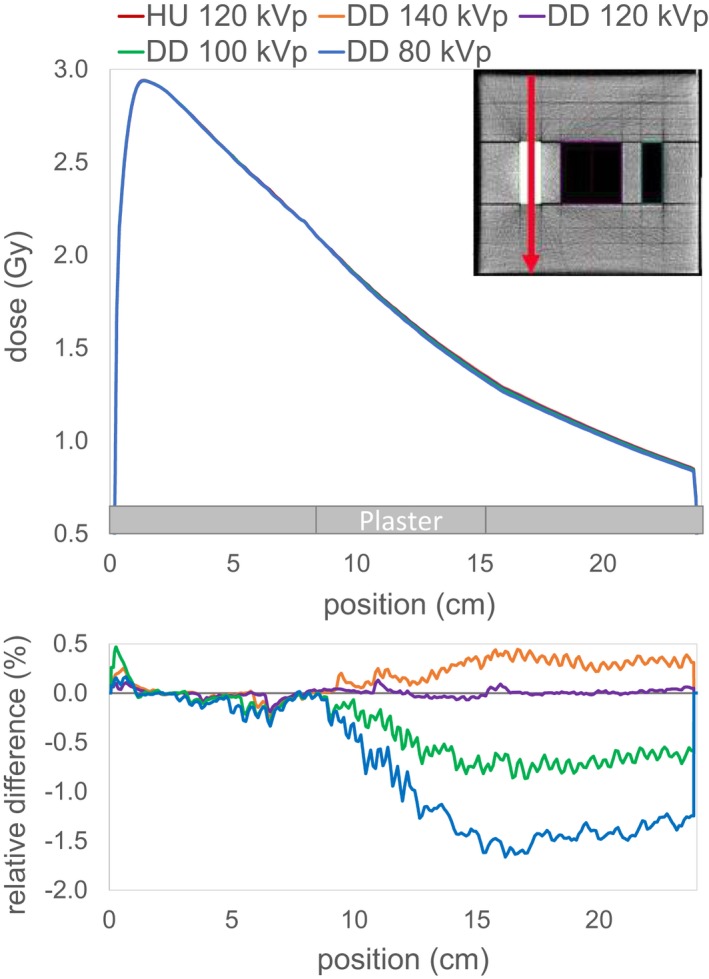
Depth dose curve through solid water and plaster indicated by the red line in the upper right corner for the single field irradiated from above for the HU and the four DD reconstructions. The lower plot shows the relative differences of the DD doses compared to the HU dose.

**Figure 6:**
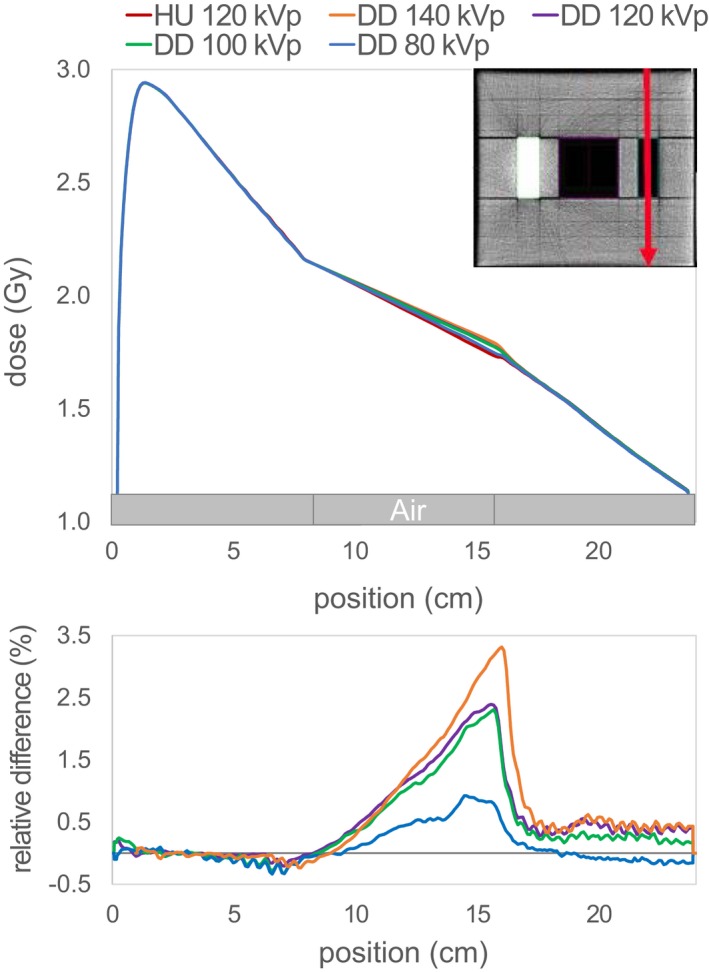
Depth dose curve through solid water and air indicated by the red line in the upper right corner for the single field irradiated from above for the HU and the four DD reconstructions. The lower plot shows the relative differences of the DD doses compared to the HU dose.

Figure 4 displays a lateral profile and Figs. 5 and 6 two depth dose curves through the center of the inserts of the IMRT phantom. The maximum dose discrepancy for plaster was −1.7% in the DD_80_ (see Fig. 5) and for air +3.3% in the DD_140_ (see Fig. 6). The dose difference in the depth dose curves for air is resolved beyond the air insert. Whereas after the plaster insert, there is still a noticeable discrepancy in the solid water after the beam traverses the plaster insert.

Despite the differences observed in the depth dose curves, there is a good agreement between the DD dose distribution and the HU reference dose for the evaluation of the 0.5% dose difference with passing rates over 98.3%. Having a closer look at the location of the failed points, it can be noticed that for the lower tube voltages more points in the plaster regions fail, while for the higher tube voltages this shifts to the air region. Because there are two plaster inserts, far more points for the DD_80_ fail resulting in a lower passing rate (98.3% in comparison to over 99%).

#### 3.A.3 Anthropomorphic geometry

**Figure 7:**
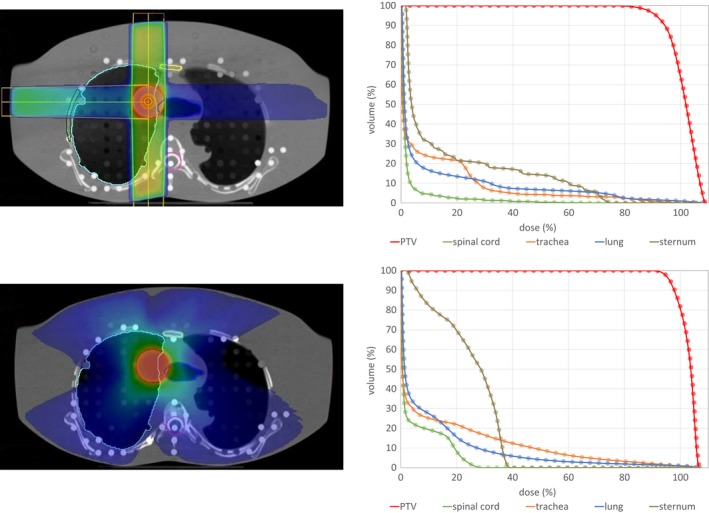
Exemplary traversal slices of the dose distribution on the left and the DHV for the thorax 3D conformal as well as the VMAT plan on the right. The dots in the DVH mark the DD results, while the solid line the HU reference DVH.

**Figure 8:**
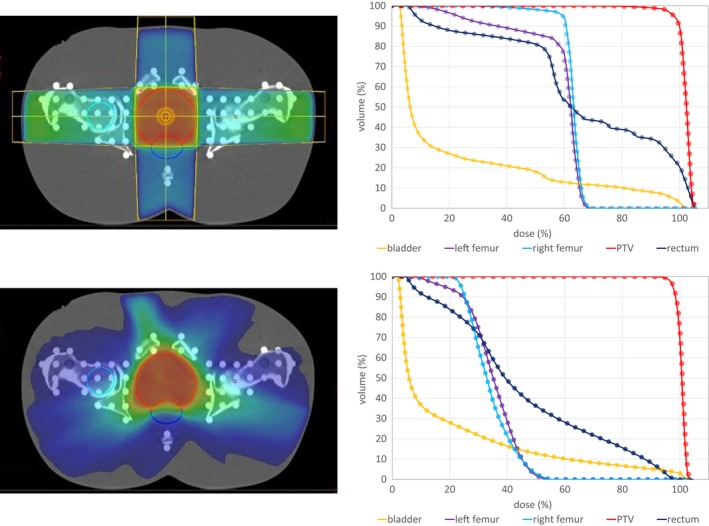
Exemplary traversal slices of the dose distribution on the left panel and the DHV for the pelvis 4‐field box as well as the VMAT plan on the right panel. The dots in the DVH mark the DD results, while the solid line the HU reference DVH.

Figures 7 and 8 show the dose distribution for the 3D conformal and VMAT plans that were optimized on the ART phantom. All DVHs for the HU and the DD dose distributions are displayed next to the transverse slice. In the DVH only minor differences can be observed, e.g., a minimal higher dose in the PTV calculated on the DD images.

The passing rates of the 0.5% dose difference for the anthropomorphic ART phantom, again, show the good agreement between the DD dose distributions when compared to the dose calculated on the HU reconstruction. The VMAT plans show an agreement of over 99.3%, while a slightly lower agreement is found in the 3D‐conformal plans (all over 98%). An exception is the 3D‐conformal thorax plan, where a large dose is deposited in the trachea leading to passing rates around 90% for 0.5% and over 98% for 1% dose difference.

As the DVH show no visible differences, the mean dose was the point chosen to evaluate. The results are shown in Table 2. In the lung as well as in the bladder, no deviation in the mean dose was found. The largest difference was observed in the trachea for the conformal thorax plan. This is due to two reasons: For one, one of the three beams aims directly at the trachea leading to a high dose in this region and additionally, the trachea is filled with air. With the results of the IMRT air cavity in mind, a higher dose has to be expected and is observed. Nevertheless, the values in Table 2 are in an order to be negligible.

**Table 2:** Mean discrepancies as well as the minimum and maximum value for the mean dose of each ROI over all DD plans on the ART phantom in the form of “Mean ∆D_mean_ [Min ∆D_mean_; Max ∆D_mean_]”.

	Mean ∆D*_mean_* (%)	
	3D	VMAT
Thorax		
Trachea	0.7 [0.0; 1.1]	0.0 [0.0; 0.0]
Sternum	0.1 [0.0; 0.6]	0.4 [0.4; 0.4]
PTV	0.2 [0.1; 0.2]	0.3 [0.3; 0.3]
Pelvis		
Femur left	‐0.1 [−0.2; 0.0]	0.2 [0.0; 0.3]
Femur right	‐0.3 [−0.3;‐0.2]	0.0 [0.0; 0.0]
Rectum	0.2 [0.1; 0.3]	0.2 [0.2; 0.2]
PTV	0.0 [−0.1; 0.1]	0.3 [0.2; 0.3]

PTV, planning target volume; ART, alderson radiation therapy; VMAT, volumetric modulated arc therapy.

### 3.B Effects of metal implants

**Figure 9:**
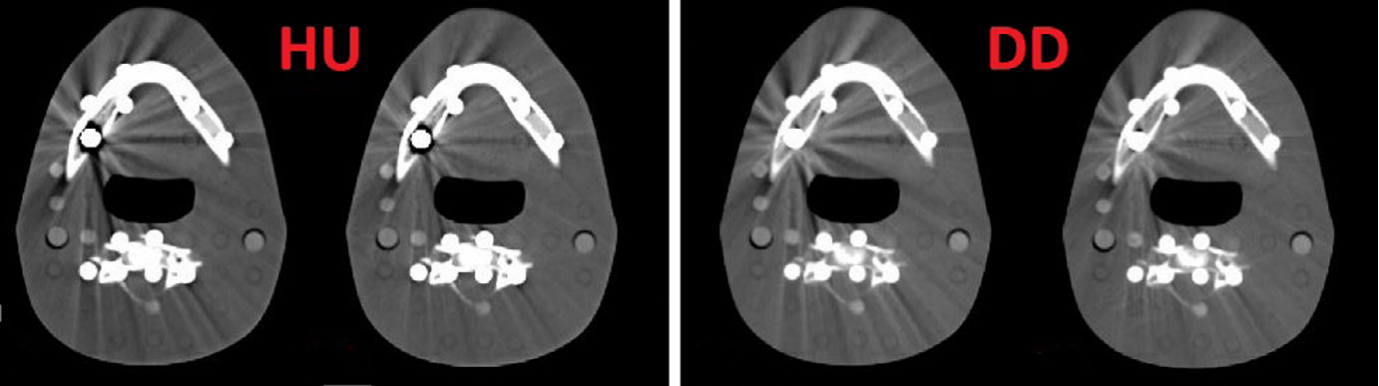
CT images with brass inserts (left on both panels) and the iMAR algorithm (right on both panels) for the HU reconstruction (left panel) and the 120 kVp DD reconstruction (right panel).

**Figure 10:**
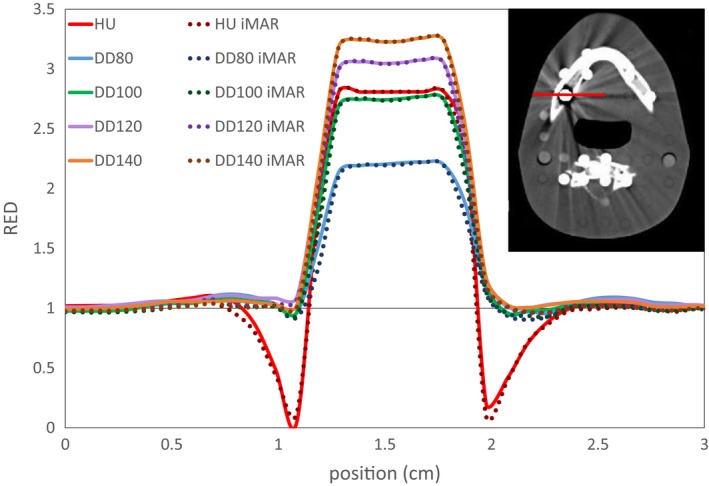
RED line through the brass insert for all reconstructions as presented with the red line on the CT image. The underestimation of the RED values around the metal can only be observed for the HU reconstruction.

Table 3 presents the mean RED values for the CBCT inserts gained with the calibration curve obtained without metal inserts (stopping at a RED value of 1.7). For both, the standard reconstruction as well as the DD reconstruction, the RED values agree with the reference given by the manufacturer for materials found in the human body and thus in the range of the calibration curve. For high density bones (or high density materials like teeth), which are not within the range of the calibration curve, a slight difference occurs and for metal, there are severe deviations. However, the differences for titanium (−25% for the HU and between −33% and −8% for the DD reconstruction) and for stainless steel (−59% HU and −64% to −50% for DD) show an equally bad RED determination of metal for all reconstructions. Figures 9 and 10 shows the same effect for the brass insert in the ART head phantom, which should have a RED value around 7. In every case, the RED is severely underestimated. Surprisingly, the DD reconstructions show no metal shadow around the insert in comparison to the HU reconstruction. No significant difference can be found between the standard and the iMAR reconstructions.

**Table 3:** Mean RED values and its standard deviation for the different inserts of the CBCT phantom for all reconstructions.

Insert	Reference	HU_120_	DD_80_	DD_100_	DD_120_	DD_140_
Lung Inhale	0.20	0.22 ± 0.02	0.23 ± 0.05	0.22 ± 0.03	0.22 ± 0.02	0.22 ± 0.02
Lung Exhale	0.50	0.50 ± 0.03	0.50 ± 0.05	0.49 ± 0.02	0.49 ± 0.02	0.49 ± 0.02
Adipose	0.95	0.94 ± 0.03	0.92 ± 0.05	0.93 ± 0.03	0.93 ± 0.02	0.93 ± 0.02
Breast	0.98	0.98 ± 0.02	0.96 ± 0.04	0.97 ± 0.03	0.97 ± 0.02	0.97 ± 0.02
Muscle	1.04	1.03 ± 0.03	1.03 ± 0.04	1.03 ± 0.03	1.04 ± 0.02	1.04 ± 0.02
Liver	1.05	1.04 ± 0.02	1.05 ± 0.04	1.05 ± 0.02	1.05 ± 0.02	1.06 ± 0.02
Bone 200	1.12	1.13 ± 0.03	1.12 ± 0.02	1.12 ± 0.02	1.11 ± 0.01	1.11 ± 0.01
Bone 800	1.46	1.49 ± 0.04	1.48 ± 0.03	1.47 ± 0.02	1.46 ± 0.02	1.46 ± 0.01
Bone 1250	1.70	1.76 ± 0.03	1.80 ± 0.04	1.77 ± 0.03	1.75 ± 0.02	1.74 ± 0.02
Titan	3.74	2.81 ± 0.01	2.51 ± 0.03	2.99 ± 0.05	3.28 ± 0.05	3.45 ± 0.06
Steel	6.92	2.81 ± 0.01	2.50 ± 0.08	2.97 ± 0.13	3.24 ± 0.16	3.41 ± 0.19

However, a calibration for high density materials could be added, diverging from the straight line from eq. (1). If this is implemented, CTs with high density materials should only be acquired with one chosen tube voltage as the metal values are not independent of the tube voltage anymore as visible in Table 3 and Fig. 10.

### 3.C Dose reduction with CARE dose

The use of the CARE kV and CARE Dose4D options allow the CT to acquire a high quality image with a potentially reduced dose exposure by choosing the kV and adapting the mAs to the given situation. While CARE Dose4D can be used with the conventional HU calibration, CARE kV can only be enabled in radiation therapy when the DD algorithm is implemented.

A comparison between the CT parameters estimated during the acquisition with and without the CARE options is given in Table 4. It shows a dose reduction via the reduction in the dose length product of 26% for the thorax scan and 46% for the pelvis scan, for the CARE Dose4D option. The pelvis scan chooses a lower voltage when CARE kV is turned on. This does not further reduce the CT dose, however, this might in some cases result in an optimized image without a higher CT Dose Index (CTDI).

**Table 4:** Comparison of the CT parameters before and after the CT options CARE kV and CARE Dose4D parameter optimization.

		Thorax				Pelvis	
	Routine	Care dose	Care kV	Routine	Care dose		Care kV
Tube voltage (kVp)	120	120	120	120	120		100
Current‐time product (mAs)	140	104	103	250	133		223
Dose‐length product (mGy·cm)	411	306	303	609	329		321
CTDIvol(32 cm)	11.3	8.4	8.4	20.0	10.8		10.5

CTDI, CT Dose Index.

**Figure 11:**
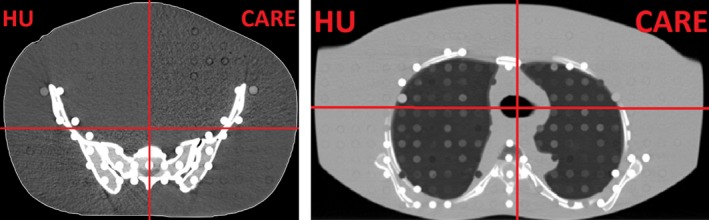
Check view image as quality check for the dose reduction with CARE. The upper left und lower right part of each view show the HU image, while the upper right and the lower left show the CARE image.

The passing rate for the dose difference between the dose calculated on these scans and the HU dose are in the same order of magnitude as all other DD scans. The image quality (see Fig. 11) was found to be sufficient.

## 4 DISCUSSION

The Siemens DirectDensity^TM^ algorithm was implemented and validated. With a simple phantom, the consistency of the RED conversion for the conventional HU to RED conversion and the Direct Density to RED conversion was tested. The results show small discrepancies that indicate that there will be discrepancies in the dose distributions. These were evaluated for different radiation modalities and setups as well as simple and also anthropomorphic geometries. A direct correlation was observed between an overestimated RED and an overestimation in dose and vice versa. Air was found to produce the biggest dose discrepancies. The highest RED difference in air was +3.3%. In general, dose was overestimated in the DD air regions in comparison to the HU dose distributions. In air, decreasing the x‐ray tube voltage lead to a decreased RED deviation and thus to a decreased dose difference. For plaster as bone substitute, a maximum RED difference of −1.7% was noticed. Here, an increased tube voltage results in an increasing agreement.

Almost all evaluated phantoms showed a good passing rate of over 99% at 0.5% dose difference (the one exception reached 98% at 1% dose difference). This matches the results of Ref. 13 where the passing rates for the gamma criteria of 1 mm and 1% were found to be greater than 99%. The results presented in this work suggest that the gamma criteria tool to evaluate the effects of the direct density dose gives a good indication but for a complete analysis it is not sufficient.

The differences for the mean dose (see Table 3) are in agreement with results from Ref. 7. Van der Heyden et al. identified that all dose deviations in the mean dose for the target volume and all ROIs were below 1%. In this work, the largest difference was found in the trachea with a deviation of 0.7% in the mean dose all other deviations were found to be below 0.4%.

Additionally, we showed that the DD reconstruction can also be used when metal implants are present. However, the metal density is dramatically underestimated in all cases.

It is advisable to focus on one tube voltage when metal implants are present and extend the calibration curve for this specific tube voltage. As this study is limited to the selected materials, a general statement about the correctness of materials foreign to the body is critical as other chemical compounds might have an influence. In these cases, however, the HU calibration is probable to also give a wrong output.

Two major benefits could result from the implementation of the DirectDensity^TM^ algorithm in the clinical routine in radiotherapy departments besides a robust workflow: The first side effect that could be exploited is the comparability with other institutions as the CT characteristics are reduced. Thus, the RED curve needed for the implementation are more alike than a standard Hounsfield lookup table. A comparison to the Siemens RED curve and to the curves used by Ritter and Van der Heyden^6^, ^7^, ^14^ shows that the slope of the calibration curve is the same in all cases (0.001, see section 2.2). However, the intercept differs by 0.006 in the maximum case, as Ritter and Van der Heyden used 1 and we fitted 0.994 as intercept. Chancing the intercept would change our results not significantly. The deviations in air would decrease while the ones for bone would slightly increase in case of low tube voltages. Thus, CTs of other institutions with DirectDensity^TM^ could be used with differences of less than 1% in the mean dose. If acceptable, this would benefit the patient by canceling the additional dose of another planning CT.

The second major benefit is the patient dose reduction by the implementation of DD with CARE kV and CARE Dose4D. This can reduce the dose significantly without reducing the image quality, allowing a more patient specific CT acquisition.

A problem that could occur with the implementation are the danger of CT scans with an unsuitable tube voltage, resulting in an unusable scan and thus a rescan with additional dose to the patient.

## 5 CONCLUSION

A CT reconstruction method for a direct translation between image values and relative electron density was implemented and found to provide sufficient image quality and dose calculation accuracy. The evaluated dose distributions showed only small differences between the dose calculated on the DirectDensity^TM^ images and on the standard HU images. The results for dose distributions calculated on CT scans containing metal implants showed a larger difference but are still in good agreement, opening up the possibility to implement the direct density reconstruction algorithm for all clinical protocols. The DirectDensity^TM^ implemented in the clinical routine allows a robust workflow while moving CT acquisition in radiotherapy to a dose optimized and thus a more personalized medicine.

## CONFLICT OF INTERESTS

We have no conflict of interest to declare.
